# Characterization of mechanisms underlying degradation of sclerotia of *Sclerotinia sclerotiorum* by *Aspergillus aculeatus* Asp-4 using a combined qRT-PCR and proteomic approach

**DOI:** 10.1186/s12864-017-4016-8

**Published:** 2017-08-31

**Authors:** Xiaojia Hu, Lu Qin, Daniel P. Roberts, Dilip K. Lakshman, Yangmin Gong, Jude E. Maul, Lihua Xie, Changbing Yu, Yinshui Li, Lei Hu, Xiangsheng Liao, Xing Liao

**Affiliations:** 1 Key Laboratory of Biology and Genetic Improvement of Oil Crops, Ministry of Agriculture, Oil Crops Research Institute, Chinese Academy of Agricultural Sciences, Wuhan, 430062 People’s Republic of China; 2 Sustainable Agricultural Systems Laboratory, Henry A. Wallace Beltsville Agricultural Research Center, USDA-Agricultural Research Service, Beltsville, MD 20705-2350 USA

**Keywords:** *Aspergillus*, Gene expression, Mycoparasitism, Proteomics, *Sclerotinia sclerotiorum*

## Abstract

**Background:**

The biological control agent *Aspergillus aculeatus* Asp-4 colonizes and degrades sclerotia of *Sclerotinia sclerotiorum* resulting in reduced germination and disease caused by this important plant pathogen. Molecular mechanisms of mycoparasites underlying colonization, degradation, and reduction of germination of sclerotia of this and other important plant pathogens remain poorly understood.

**Results:**

An RNA-Seq screen of Asp-4 growing on autoclaved, ground sclerotia of *S. sclerotiorum* for 48 h identified 997 up-regulated and 777 down-regulated genes relative to this mycoparasite growing on potato dextrose agar (PDA) for 48 h. qRT-PCR time course experiments characterized expression dynamics of select genes encoding enzymes functioning in degradation of sclerotial components and management of environmental conditions, including environmental stress. This analysis suggested co-temporal up-regulation of genes functioning in these two processes. Proteomic analysis of Asp-4 growing on this sclerotial material for 48 h identified 26 up-regulated and 6 down-regulated proteins relative to the PDA control. Certain proteins with increased abundance had putative functions in degradation of polymeric components of sclerotia and the mitigation of environmental stress.

**Conclusions:**

Our results suggest co-temporal up-regulation of genes involved in degradation of sclerotial compounds and mitigation of environmental stress. This study furthers the analysis of mycoparasitism of sclerotial pathogens by providing the basis for molecular characterization of a previously uncharacterized mycoparasite-sclerotial interaction.

**Electronic supplementary material:**

The online version of this article (doi:10.1186/s12864-017-4016-8) contains supplementary material, which is available to authorized users.

## Background

*Sclerotinia sclerotiorum* (Lib.) de Bary is an important soilborne sclerotial pathogen that causes diseases on over 400 plant species from at least 75 families throughout the world including many major agronomic crops [1, 2]. Application of fungicides is the primary method of disease control but control is problematic due to the residence of sclerotial resting structures in soil; these sclerotia being the source of initial inoculum for disease. Large amounts of fungicide are often required for soil pathogens and control can be inconsistent [3]. Loss of fungicide effectiveness due to the development of fungicide resistance in pathogen populations is also a concern [4–6]. Other disease control measures such as crop rotation can be ineffective due to the wide host range of *S. sclerotiorum* and the long persistence of recalcitrant sclerotial resting structures in soil [7]. Breeding for resistance has been hampered in some cases by a limited gene pool and the fact that resistance can be governed by multiple plant genes [8–10].

The use of mycoparasites as biological control agents is an alternative strategy for control of *S. sclerotiorum* [3, 11, 12]. These mycoparasites are applied to the field and expected to colonize and kill or weaken sclerotia resulting in the reduction of initial inoculum. Sclerotia, such as those produced by *S. sclerotiorum*, are hyphal aggregates that consist of a pigmented rind, a thin-walled cortex, and a large central medulla [13–15]. The cell walls of these hyphae consist primarily of glucan polymers; these glucans being predominantly ß-1,3-linked polymers but ß-1,6-; mixed ß-1,3- and ß-1,4-; α1,3-; and α1,4-linked glucans have been found [16]. Chitin, a homopolymer of ß-1,4-linked N-acetylglucosamine, is structurally important and can comprise as much as 10–20% of the cell wall. Additionally, fungal cell walls have glycoprotein, containing mannose and sometimes galactose moieties, interwoven within the chitin and glucan polymers [16]. Pigmentation in the sclerotial rind of *S. sclerotiorum* is due to melanin, in this case a polymer of dihydroxynapthalene [17]. In addition to the daunting physical barrier due to the complexity of these interwoven polymers, mycoparasites are likely to encounter potentially stressful conditions as *S. sclerotiorum* produces toxic phenolic compounds and fatty acids [18], reactive oxygen species [19], organic acids such as oxalic acid, and various enzymes capable of producing toxic products [20].

There has been some success in developing mycoparasites for production agriculture but grower acceptance of biological control products in general has been slowed by their inconsistent performance [21, 22]. For example, inconsistent performance has been reported in field trials with *Coniothryium minitans* in comparison with fungicide applications [4, 23]; *C. minitans* being the biological control agent in a commercial biological control product marketed for control of *S. sclerotiorum*. Molecular approaches have been used in recent years to more thoroughly understand the fungal – fungal interactions involved in mycoparasitic biological control strategies in attempts to improve their effectiveness; these molecular studies being largely limited to work with *Trichoderma* sp. and *C. minitans* [24–28]. Here we use transcriptomic, qRT-PCR, and proteomic approaches to characterize the mycoparasitic interaction between *Aspergillus aculeatus* isolate Asp-4 and sclerotia of *S. sclerotiorum*, expanding information regarding mycoparasitism of sclerotia with this study of an additional mycoparasitic fungal – sclerotia interaction. Isolate Asp-4 effectively inhibited germination of sclerotia of *S. sclerotiorum* in the field and reduced incidence of disease caused by this pathogen on oilseed rape [29, 30]. Isolate Asp-4 was particularly effective in colonizing and degrading tissue of sclerotia of *S. sclerotiorum*, causing a 60% reduction in the mass of sclerotia over a 72 h period in vitro [30].

## Methods

### Fungal isolate

*Aspergillus aculeatus* isolate Asp-4 was obtained from the culture collection of the Plant Protection Laboratory, Oil Crops Research Institute, Wuhan, People’s Republic of China where it was stored long-term in 20% glycerol at −80 °C. Isolate Asp-4 was initially isolated from soil from a research plot at the Oil Crops Research Institute [31] and identified using morphological characteristics and sequence of ITS1 and ITS4 regions from rDNA. Unless indicated otherwise isolate Asp-4 was cultured in potato dextrose broth (PDB) or potato dextrose agar (PDA) plus 50 μg/ mL hygromycin as this isolate was naturally resistant to that level of hygromycin. Isolate Asp-4 is also held in the Agricultural Culture Collection of China (Beijing) as ACCC 32502.

### RNA isolation from Asp-4, cDNA library preparation, and transcriptome sequencing

RNA was isolated from Asp-4 grown on a medium containing sclerotia of *S. sclerotiorum*. For this medium sclerotia of *S. sclerotiorum* were collected from the field and verified through morphological examination and 18S rDNA sequencing. DNA was extracted from sclerotia using standard procedures and the primers 18SrDNA-for (5′ TCCGTAGGTGAACCTGCGG 3′) and 18SrDNA-rev (5′ TCCTCCGCTTATTGATATGC 3′) used to amplify 18S DNA for sequencing [32]. Sclerotia verified as *S. sclerotiorum* were dried 3 days at 25 to 28 °C, ground with mortar and pestle, passed through a 20 mesh sieve, autoclaved at 121 °C for 30 min in 0.9% NaCl in glass petri dishes, and autoclaved again at 121 °C for 30 min. Petri dishes contained 5 g ground sclerotia in 15 mL 0.9% NaCl. Cellophane, containing 0.2 μm holes (Cat. No. 11–40-0, Shanghai Biological Technology Co., Ltd., Shanghai) was autoclaved at 121 °C for 30 min in separate glass petri dishes and placed on top of the twice-autoclaved sclerotial material prior to inoculation with Asp-4. For the control, RNA was isolated from Asp-4 grown on sterile cellophane covering PDA.

For inoculation of these media, a 0.1 mL spore suspension of Asp-4 in 0.9% NaCl (≥ 1 × 10^3^ spores/ mL) from the −80 °C freezer stock was placed on a PDA starter plate and incubated at 28 °C. A 0.1 mL spore suspension of Asp-4 in sterile 0.9% NaCl (≥ 1 × 10^3^ spores/ mL) obtained from this starter plate was transferred onto the sterile cellophane covering ground sclerotia in glass petri dishes or covering PDA in the control. Mycelia of isolate Asp-4 cultured for 48 h at 28 °C on five petri dishes containing crushed sclerotia were collected and combined and mycelia from Asp-4 cultured 48 h at 28 °C on five petri dishes containing the PDA control were collected and combined. Mycelia from both treatments were simultaneously snap-frozen in liquid N_2_ and stored at −80 °C until used.

Mycelia in liquid N_2_ were ground with a mortar and pestle prior to RNA isolation with TRIZOL [33]. RNA quality was determined with a NanoPhotometer® spectrophotometer (IMPLEN, Inc., Westlake Village, CA). Integrity and quantity of RNA was assessed using an RNA Nano 6000 Assay Kit with the Agilent Bioanalyzer 2100 system according to the manufacturer’s instructions (Agilent Technologies, Santa Clara, CA). A total of 3 μg high-quality RNA from each treatment was used for cDNA synthesis using the NEBNext® Ultra™ RNA Library Prep Kit for Illumina® (New England Biolabs, Ipswich, MA) following the manufacturer’s recommendations. Library quality was determined using the Agilent Bioanalyzer 2100 system. Clustering of the index-coded samples was performed on a cBot Cluster Generation System using the TruSeq PE Cluster Kit v3-cBot-HS (Illumina, Inc., San Diego, CA) according to the manufacturer’s instructions. After cluster generation, library preparations were sequenced on an Illumina HiSeq 2000 platform and paired-end reads generated.

### RNA-Seq transcriptome analysis, assembly, and functional annotation

Raw data (raw reads) in fastq format were processed through in-house perl scripts to clean data by removing reads containing adapter, reads containing poly-N, and reads of low quality. All downstream analyses were performed with the clean data. Q20, Q30, GC-content, and sequence duplication levels were calculated. Transcriptome assembly, based on left.fq and right.fq, was performed using Trinity (ver. 2012–10-05) [34] with min_kmer_cov set to 2 and all other parameters set to default. Gene function was annotated based on Nr (ver. 2014.1.25; National Center for Biotechnology Information [NCBI]), Nt (ver. 2014.1.25; NCBI), Pfam (ver. 27.0:2013.5.22), KOG/COG (ver. 2013:12.5), Swiss-Prot (ver. 2014.1.25), KO (kaas_sa22; KEGG Ortholog), and GO (GO Blast2GO ver. 2.5; Gene Ontology) databases [35, 36]. Gene Ontology enrichment analysis of the differentially expressed genes (DEGs) was conducted using the GOseq-R-package (GO Blast2GO ver. 2.5)-based Wallenius non-central hyper-geometric distribution [37]. KEGG pathway analysis was used to assign the all-unigenes to biological pathways.

For the sequenced library, read counts were adjusted with the edgeR program package (ver. 3.0.8) [38] through one scaling normalized factor. Differential expression analysis was performed using the DEGseq R package (ver. 1.10.1) [39]. Poisson distribution was used for *p* value calculation and *p* value was adjusted using q value [40]; q value <0.005, and log2 (fold change) > 1 were used as the threshold for significant differential expression.

### Analysis of differential gene expression by qRT-PCR

Genes were selected from the transcriptome data for analysis of differential expression by qRT-PCR. RNA was isolated using RNAiso (Takara, Shiga, Japan) from Asp-4 grown on crushed sclerotia or PDA as described above for various times and treated with RNAse-free DNase I (Thermo Fisher Scientific, Inc., Waltham, MA). First strand cDNA was synthesized from total RNA with an oligo(dT)18 primer in a 20 μL reaction using an M-MLV Reverse Transcriptase according to the manufacturer’s instructions (Thermo Fisher Scientific, Inc., Waltham, MA). The synthesized cDNA was diluted and used as a template for real-time PCR reactions using a Real-Time PCR system (CFX Connect™, Bio-Rad, Inc.). Each reaction (20 μL) contained 10 μL of Power SYBR® Green PCR Master Mix (Applied Biosystems®, Foster City, CA), forward and reverse primers, cDNA template, and nuclease-free water. Primer Premier 5.0 (PREMIER Biosoft, Palo Alto, CA) was used for primer design (listed in Additional file 1: Table S1). PCR conditions were: 3 min at 95 °C (1 cycle), 10 s at 95 °C followed by 20 s at 55 °C (45 cycles), and a melting curve ramping from 65 to 95 °C with an increasing temperature of 0.5 °C. *act* and *cox5* transcripts, encoding actin and cytochrome c oxidase subunit V, were used as internal references to normalize RNA in each reaction. Gene expression levels were calculated from the threshold cycle according to the 2^-ΔΔCT^ method. All samples were analyzed in two independent experiments with three replicates.

### Protein extraction from Asp-4 mycelia for 2-D gel electrophoresis

Mycelia of isolate Asp-4, cultured 48 h on crushed sclerotia or cultured 48 h on the PDA control, were collected separately, and snap-frozen in liquid N_2_ exactly as for the RNA transcriptome experiment. For the second experiment the same suspension of isolate Asp-4 that was used in the first experiment was transferred to the glass petri dishes containing sclerotia or the PDA control 16 h later. Frozen mycelia were ground in liquid N_2_ in a pre-cooled mortar and pestle and extracted as per Hurkman and Tanaka [41] in Tris-saturated phenol and extraction buffer (0.7 M sucrose, 0.1 M KCl, 50 mM EDTA, 0.5 M Tris-HCl, pH 7.5, 2% β-mercaptoethanol, and 1 mM PMSF). The protein in the phenol phase was precipitated with 0.1 M ammonium acetate in methanol followed by incubation at −20 °C overnight. Precipitated protein was collected by centrifugation. The protein pellet was washed twice with pre-cooled methanol, twice with pre-cooled acetone, vacuum-dried, and stored at −80 °C until used.

### Two-dimensional gel electrophoresis (2-DGE)

Protein pellets were redissolved in rehydration buffer and protein concentrations determined with the Bradford assay [42]. Immobilized pH strips (pH 4–7, nonlinear, 24 cm, Immobiline Drystrip, BioRad, Hercules, CA) were rehydrated in protein solution for 12 h at 20 °C and isoelectric focusing (IEF) carried out using an Ettan IPGphor 3 isoelectric focusing system (GE Healthcare, Wauwatosa, WI) at 300 V (0.5 h, step), 700 V (0.5 h, step), 1500 V (1.5 h, step), 9000 V (3 h, gradient) and 9000 V (5 h, step) for a total of 64 kVh. Focused strips were equilibrated 15 min with equilibration buffer (100 mM DTT, 6 M urea, 30% *w/v* glycerol, 2% SDS, 50 mM Tris-HCl, pH 8.8, 0.002% bromophenol blue) and then for 15 min in the same equilibration buffer amended with 250 mM iodoacetamide. The second dimension was performed in 12.5% sodium dodecylsulfate polyacrylamide gels (SDS-PAGE) on an Ettan DALTsix unit (GE Healthcare) according to the manufacturer’s recommendations until the tracking dye reached the bottom of the gel. Gels were fixed and stained using Coomassie Brilliant Blue G (Sigma Chemical Co., St. Louis, MO) and silver stain. Stained gels were scanned and image analysis carried out with Image Master 2D Platinum 5.0 software (GE Healthcare). After scanning, individual protein spots were assigned a grey scale value. A protein in the sclerotia treatment was considered differentially expressed if its grey scale value was two-fold greater than or less than the corresponding protein in the PDA control treatment. Two replicate 2-DGE gels were run for each of the two experiments.

### In-gel digestion and protein identification by mass spectrometric analysis

Differentially expressed protein spots were excised manually from the gels, destained, and digested with trypsin. After digestion, peptides were extracted with 60 μL extract solution containing 2.5% trifluoroacetic acid and 90% acetonitrile, vacuum-dried, dissolved in 1.5 μL matrix solution, and spotted onto a MALDI-TOF target plate. Mass spectrometric analysis of peptides from gel spots was performed using an Ultraflex III TOF/TOF mass spectrometer (Bruker Daltonics, Freemont, CA) using a UV laser with a wavelength of 355 nm and operated at a 200 Hz repetition rate. All acquired spectra of samples were identified using flexAnalysis (Bruker Daltonics), and the MS/MS data was analyzed using BioTools software (Bruker Daltonics) to search for proteins in the NCBI database with the following search parameters: protein molecular mass 800 to 4000 Da, MS tolerance set at 500 ppm, MS/MS tolerance of 0.5 Da.

### Validation of differential protein expression by qRT-PCR

Genes were inferred from the proteomic data for validation of differential expression by qRT-PCR. RNA preparation, primer design PCR cycling conditions, and analysis were as described for the qRT-PCR experiments. Primers used are listed in Additional file 2: Table S2. The same mycelia preparations used for the above protein extraction were also used for the RNA preparations. All samples were analyzed in at least two independent experiments with three replicates.

## Results

### RNA-seq transcriptome sequencing, sequence assembly, and functional annotation of *Aspergillus aculeatus* Asp-4

Transcriptomes of isolate Asp-4 grown on ground sclerotia of *S. sclerotiorum* in 0.9% NaCl for 48 h at 28 °C and on the PDA control were determined from one experiment. Samples from each treatment produced over 2.3G raw data, had a Q20 of over 95%, a Q30 of over 87%, and a sequencing error rate lower than 0.05% indicating that the sequencing quality was suitable for further analysis. The samples were 56% GC. Using the Trinity de novo assembly method, short sequence reads were assembled into 55,880 transcripts and these transcripts used for cluster and assembly analyses. A total of 37,278 unigenes were obtained of which 19,138 (51.34%) were longer than 500 bp (Additional file 3: Fig. S1a). All 37,278 unigenes were searched against Nr, Nt, Swiss-Prot, KEGG, and COG databases. These analyses revealed that 27,926 unigenes (74.91%) had significant matches in the Nr database, 10,109 unigenes had significant matches in the Nt database (27.11%), and 17,361 unigenes (46.57%) had significant matches in the SwissProt database. Gene ontology (GO) assignments were used to classify the functions of Asp-4 unigenes in GO terms (Additional file 3: Fig. S1b). Greater than 10% of unigenes in the biological process category were labeled with the terms biological regulation, cellular process, establishment of localization, localization, metabolic process, regulation of biological process, and response to stimulus. Greater than 10% of unigenes in the cell component category were labeled with the terms cell, cell part, macromolecular complex, membrane, membrane part, organelle, and organelle part. Finally, greater than 10% of unigenes in the molecular function category were labeled with the terms binding and catalytic activity. KEGG pathway tools were used to further identify biological pathways. The top 31 KEGG pathways are shown in Additional file 4: Fig. S2; with the largest percentage of unigenes (> 10%) classified under carbohydrate metabolism, amino acid metabolism, and translation. A large number of unigenes (5% to 10%) were also classified under transport and catabolism; signal transduction; folding, sorting, and degradation; energy metabolism; lipid metabolism; and metabolism of cofactors and vitamins.

### Transcriptional response of *Aspergillus aculeatus* Asp-4 during growth on sclerotia of *S. sclerotiorum*

RNA-Seq data from Asp-4 grown on sclerotia of *S. sclerotiorum* for 48 h was compared with RNA-Seq data from Asp-4 grown on PDA for 48 h to screen for differentially expressed genes. A log2-fold difference in expression between treatments was used as the threshold. There were 997 genes up-regulated in the treatment where Asp-4 was grown on sclerotia as the nutrition source relative to Asp-4 grown on PDA and 777 genes down-regulated (Additional file 5: Tables S3 and Additional file 6: Table S4). Up-regulated genes included those from the following groups: carbohydrate metabolism, amino acid metabolism, energy metabolism, cell wall metabolism, regulation, heat shock protein, and transport. Down-regulated genes in the treatment where Asp-4 was grown on sclerotia as the nutrition source relative to Asp-4 grown on PDA included those involved in carbohydrate metabolism, amino acid metabolism, and transport.

A time course experiment was conducted using qRT-PCR to determine changes in expression of 27 select Asp-4 genes, identified in the RNA-Seq screen experiment, over the initial 48 h of colonization of sclerotial material (Table 1). The experiment was performed twice. Mean values for expression from two experiments (*n* = 2) were only considered in the analysis when the mean was greater than the standard deviation of the mean.

**Table 1:** Relative expression values of genes during colonization of autoclaved sclerotia of *Sclerotinia sclerotiorum* by *Aspergillus aculeatus* Asp-4

Putative function	Gene ID	Time after inoculation (Mean +/− SD)			
		12 h	24 h	36 h	48 h
Glucanases					
Avicelase III; [*Aspergillus aculeatus*]	Comp15820_c0	1.1 ± 0.2	5.1 ± 1.2	9.0 ± 0.2	885.0 ± 122.0
Cell wall glucanase; [*Aspergillus niger* CBS 513.88]	Comp10145_c0	1.1 ± 0.1	1.3 ± 0.3	1.3 ± 0.2	6.7 ± 6.2
Endoglucanase-4; [*A. niger* CBS 513.88] > gi|134,080,248	Comp6428_c0	1.2 ± 0.3	(21.8 ± 22.1)	2.8 ± 0.8	(382.3 ± 473.2)
1,4-ß-D-glucan cellobiohydrolase A	Comp13421_c0	0 ± 0	1.7 ± 1.0	0 ± 0	70.3 ± 62.9
1,4-ß-D-glucan cellobiohydrolase B	Comp17477_c0	1.3 ± 0.5	(69.0 ± 81.7)	3.3 ± 0.9	(4173.7 ± 4754.0)
1,4-ß-D-glucan cellobiohydrolase C; [*A. niger* CBS 513.88]	Comp19016_c0	1.0 ± 0.1	(146.1 ± 157.3)	25.6 ± 15.8	(64,056.3 ± 87,096)
ß-glucosidase G [*A. niger* CBS 513.88]	Comp16375_c2	3.0 ± 0.0	3.66 ± 0.0	1.8 ± 0.2	48.2 ± 29.2
Other polysaccharide depolymerases					
Mannan endo-1,4-β-mannosidase A	Comp7543_c0	1.9 ± 1.2	22.8 ± 8.2	16.0 ± 2.7	1167.2 ± 642.8
Pectin lyase F; [*Aspergillus oryzae* RIB40] > gi|391,874,352	Comp10369_c0	11.7 ± 1.3	4.3 ± 1.3	1.6 ± 0.8	(33.1 ± 35.1)
Endo-α-1,4 polygalactosaminidase; [*A. niger* CBS513.88]	Comp10792_c0	4.1 ± 0.6	3.0 ± 1.1	1.2 ± 0.3	20.9 ± 9.7
Rhamnogalacturonate lyase A	Comp15535_c0	4.4 ± 0.5	3.0 ± 1.1	1.2 ± 0.3	20.0 ± 10.9
Endo-arabinase; [*A. niger* CBS 513.88]	Comp15955_c0	1.1 ± 0.2	3.4 ± 0.2	3.8 ± 0.2	154.6 ± 153.0
Lipases					
Lipase; [*A. niger* CBS 513.88] > gi|134,083,043|e	Comp10309_c0	5.8 ± 1.5	6.3 ± 0.8	5.4 ± 1.4	120.2 ± 94.6
Phospholipase; [*A. niger* CBS 513.88]	Comp19032_c0	1.5 ± 0.2	1.6 ± 0.4	1.7 ± 0.9	6.0 ± 1.9
Environmental management					
Heat shock trehalose synthase; [*A. niger* CBS 513.88] > gi|134,084,204|	Comp13560_c0	1.1 ± 0.2	11.1 ± 3.6	75.4 ± 0.7	4333.0 ± 2354.2
30kD heat shock protein; [*A. niger* ATCC 1015]	Comp17224_c0	4.9 ± 3.7	3.3 ± 3.3	3.1 ± 1.7	73.3 ± 36.7
Protease inhibitor; [*Talaromyces marneffei* ATCC 1822]	Comp16974_c0	54.0 ± 13.9	10.5 ± 4.6	11.4 ± 6.5	165.2± 94.7
Oxylate decarboxylase OxdC; [*Bacillus subtilis* (strain 168)]	Comp22183_c0	1.0 ± 0.0	1.8 ± 0.2	5.5 ± 0.2	13.3 ± 3.0
CDR ABC transporter; [*Byssochlamys nivea*]	Comp16806_c0	20.6 ± 6.9	9.6 ± 0.6	4.4 ± 1.5	(166.4 ± 175.2)
ABC multidrug transporter; [*A. oryzae* RIB40]	Comp15840_c0	1.8 ± 1.1	692.6 ± 203.7	877.9 ± 347.1	915.9 ± 83.8
Regulation					
RNA polymerase II transcription factor [*A. niger* CBS 513.88] > gi|134,074,447|	Comp7059_c0	1.2 ± 0.3	1.7 ± 0.1	2.7 ± 1.5	(346.1 ± 354.4)
Fungal specific transcription factor domain protein [*A. niger* CBS 513.88]	Comp12975_c0	1.8 ± 1.1	(10.6 ± 12.2)	10.0 ± 6.4	3428.3 ± 3025.3
Transcription factor TFIID complex 145 kDa subunit [*Aspergillus kawachii* IFO 4308]	Comp14528_c0	1.1 ± 0.1	1.6 ± 0.3	1.4 ± 0.1	49.8 ± 32.6
C6 transcription factor; [*A. niger* CBS 513.88]	Comp15729_c0	1.0 ± 0.0	1.3 ± 0.3	2.0 ± 0.2	87.3 ± 74.4
GATA transcription factor LreB; [*Aspergillus fumigatus* A1163]	Comp16275_c7	1.0 ± 0.1	2.8 ± 0.0	3.0 ± 1.1	35.1 ± 23.1
Transcriptional regulator Ngg1; [*A. fumigatus* Af293] > gi|66,847,840|	Comp15147_c0	1.7 ± 0.5	1.7 ± 1.0	3.6 ± 0.9	(124.0 ± 127.3)
Sensor histidine kinase; [*Aspergillus clavatus* NRRL	Comp15130_c0	1.2 ± 0.2	2.3 ± 0.9	2.8 ± 0.0	39.6 ± 11.2

Gene expression levels were calculated from the threshold cycle according to the 2^-ΔΔCT^ method. Values are the mean of two experiments (*n* = 2), each with three replicates, with standard deviation. *a* *ct* and *cox5* transcripts, encoding actin and cytochrome c oxidase subunit V, were used as internal references to normalize RNA in each reaction. SD, standard deviation. Values in parentheses were considered too variable to be used in the analysis as the standard deviation was greater than the mean

Carbohydrate polymer degrading enzmes which potentially can degrade glycoprotein in fungal cell walls (mannan endo-1,4-β-mannosidase A, comp7543_c0) or which act on pectic material (pectin lyase F, comp10369_c0; endo-α-1,4 polygalactoseaminidase, comp10792_c0; rhamnogalacturonate lyase A, comp15535_c0) and thus have been correlated with degradation of fungal cell walls [43, 44], were studied. Genes for all enzymes with the exception of pectin lyase A had levels of expression at 48 h that were greater than that at 12 h (Table 1). Notably, expression of mannan endo-1,4-β-mannosidase A was 630-fold greater at 48 h than at 12 h. Expression of the gene for endo-arabinase (comp15955_c0), another carbohydrate depolymerase, increased slightly from 12 h to 36 h and expression levels at 48 h were 150-fold greater than at 12 h but highly variable (Table 1).

Expression of genes for three endoglucanases (Avicelase III, comp15820_c0; cell wall glucanase, comp10145_c0; endoglucanase-4, comp6428_c0), three 1,4-β-D-glucan cellobiohydrolases (A, comp13421_c0; B, comp17477_c0; C, comp19016_c0), and ß-glucosidase G (comp16375_c2) was also studied in the time course experiment (Table 1). A notable increase in expression of the endoglucanase Avicelase III was detected with expression at 48 h being 885-fold greater than at 12 h. There were also substantial increases in expression of cellobiohydrolase A and C genes and the ß-glucosidase G gene. Expression of all of these genes increased over the 48 h experiment. Lipids have been detected in sclerotia from *S. sclerotiorum* [45, 46]. Genes associated with degradation of lipid were expressed during colonization of sclerotial material by Asp-4 as genes for lipase (comp10309_c0) and phospholipase (comp19032_c0) increased in expression over the 48 h experiment with levels of expression at 48 h being 20-fold and 4-fold greater, respectively, than those at 12 h (Table 1).

Expression of Asp-4 genes potentially involved in adapting to environmental stress conditions was also typically mani-fold greater at 48 h after initiation of colonization of the sclerotial material than at 12 h (Table 1). Notably, expression of the gene for heat shock trehalose synthase (comp13560_c0) was 3768-fold greater at 48 h than at 12 h after initiation of colonization. Expression of the 30kD heat shock protein gene (comp17224_c0) was 15-fold greater at 48 h than at 12 h. Finally, a protease inhibitor (comp16974_c0) and oxylate decarboxylase (comp22183_c0) were 3-fold and 13-fold greater, respectively, at 48 h than at 12 h after initiation of colonization. Expression of an ABC multidrug transporter (comp16806_c0) was also greater at 48 h than at 12 h, with the level of expression 509-fold greater at 48 h. These transporters transport compounds like toxic drugs and other compounds [47, 48]. Expression of a CDR ABC transporter (comp15840_c0) decreased from 12 h to 36 h and was too variable to be used in the analysis at 48 h.

There was substantial up-regulation of four of the six transcription factors (RNA polymerase II transcription factor, comp7059_c0; fungal specific transcription factor domain protein, comp12975_c0; transcription factor TFIID complex 145 kDa subunit, comp14528_c0; C6 transcription factor, comp15729_c0; GATA transcription factor LreB, comp16275_c7; transcriptional regulator Ngg1, comp15147_c0) studied and a sensor histidine kinase (sensor histidine kinase/response regulator, comp15130_c0) of Asp-4 during the initial 48 h of colonization of sclerotial material (Table 1). Expression at 48 h of all of these genes except RNA polymerase II transcription factor and transcriptional regulator Ngg1was at least 33-fold greater than at 12 h, the greatest relative increase being 1936-fold by the fungal specific transcription factor domain protein. Notably, expression of C6 transcription factor, which is associated with an oxidative stress response and the production of an anti-predation metabolite in *Aspergillus* spp. [49, 50], was 87-fold greater at 48 h than at 12 h. The expression of RNA polymerase II transcription factor and transcriptional regulator Ngg1 genes was fairly constant from 12 h to 36 h and too variable between experiments at 48 h to be used in the analysis. Transcriptional regulator Ngg1 is involved in glucose repression of Gal4p-regulated genes [51].

### Transcriptional response of *Aspergillus aculeatus* Asp-4 during growth on PDA

Two qRT-PCR time course experiments were also conducted to determine changes in expression of select Asp-4 genes over the initial 48 h of growth by this mycoparasite on PDA (Table 2). These experiments were conducted concurrently with the above time course experiments to determine if expression of genes on the sclerotial material was unique or possibly just due to growth on complex polymeric substrates. Mean values for expression were only considered in the analysis when the mean (*n* = 2) was greater than the standard deviation of the mean.

**Table 2:** Relative expression values of genes by *Aspergillus aculeatus* Asp-4 during growth on PDA

Putative function	Time after inoculation (Mean +/− SD)			
	12 h	24 h	36 h	48 h
Glucanases				
Avicelase III	8.9 ± 4.0	(64.0 ± 89.0)	566.0 ± 354.3	(124.0 ± 146.3)
Cell wall glucanase	1.9 ± 0.0	3.0 ± 0.8	6.7 ± 0.8	3.9 ± 0.3
Endoglucanase-4	1.9 ± 0.3	8.2 ± 5.3	71.0 ± 12.8	33.2 ± 5.6
1,4-ß-D-glucan cellobiohydrolase A	0.0 ± 0.0	0.0 ± 0.0	0.0 ± 0.0	0.0 ± 0.0
1,4-ß-D-glucan cellobiohydrolase B	2.6 ± 0.2	30.1 ± 24.9	212.7 ± 159.5	(128.6 ± 149.4)
1,4-ß-D-glucan cellobiohydrolase C	4.4 ± 1.4	(140.5 ± 140.3)	(2276.8 ± 2527.7)	286.9 ± 148.4
ß-glucosidase G	1.6 ± 0.8	5.4 ± 2.9	30.2 ± 28.0	14.3 ± 3.8
Other polysaccharide depolymerases				
Mannan endo-1,4-β-mannosidase A	48.9 ± 19.1	42.0 ± 1.8	145.2 ± 98.7	(36.1 ± 36.1)
Pectin lyase F	6.1 ± 4.1	4.3 ± 2.2	(15.6 ± 17.5)	2.1 ± 0.1
Endo-α-1,4 polygalactosaminidase	1.5 ± 0.5	3.3 ± 1.0	6.7 ± 2.7	2.9 ± 0.0
Rhamnogalacturonate lyase A	1.5 ± 0.5	3.5 ± 1.1	5.7 ± 2.0	2.9 ± 0.2
Endo-arabinase	2.0 ± 0.4	6.1 ± 5.5	99.8 ± 87.5	25.9 ± 3.2
Lipases				
Lipase	1.8 ± 1.2	13.9 ± 4.9	65.4 ± 61.8	17.8 ± 1.8
Phospholipase	1.0 ± 0.0	2.1 ± 1.3	4.9 ± 1.4	5.2 ± 1.0
Environmental management				
Heat shock trehalose synthase	1.7 ± 0.1	50.0 ± 40.9	(931.1 ± 1030.7)	425.8 ± 78.0
30kD heat shock protein	9.3 ± 1.7	17.1 ± 3.9	92.9 ± 1.7	256.8 ± 21.6
Protease inhibitor	10.5 ± 1.7	3.2 ± 0.6	9.2 ± 1.7	1.3 ± 0.4
Oxylate decarboxylase OxdC	2.5 ± 0.1	13.2 ± 0.0	26.0 ± 10.1	12.9 ± 2.8
CDR ABC transporter	1.1 ± 0.2	7.1 ± 6.7	(76.1 ± 80.3)	14.4 ± 0.4
ABC multidrug transporter	2.0 ± 0.5	371.8 ± 211.1	1487.8 ± 470.7	9339.8 ± 1873.5
Regulation				
RNA polymerase II transcription factor	4.0 ± 0.8	26.1 ± 8.4	190.8 ± 171.8	61.3 ± 7.6
Fungal specific transcription factor domain protein	1.7 ± 0.2	78.3 ± 47.2	(383.7 ± 449.8)	486.6 ± 282.4
Transcription factor TFIID complex 145 kDa subunit	1.8 ± 0.5	6.9 ± 3.6	15.1 ± 11.5	27.8 ± 6.3
C6 transcription factor	2.2 ± 0.4	14.2 ± 5.0	52.6 ± 44.0	21.8 ± 0.6
GATA transcription factor LreB	3.4 ± 1.1	7.0 ± 5.1	54.5 ± 20.0	43.2 0.3
Transcriptional regulator Ngg1	2.8 ± 1.0	55.4 ± 33.2	199.8 ± 104.4	172.9 ± 3.0
Sensor histidine kinase	6.4 ± 0.2	(6.3 ± 6.2)	68.3 ± 50.9	22.5 ± 3.0

Gene expression levels were calculated from the threshold cycle according to the 2^-ΔΔCT^ method. Values are the mean of two experiments (*n* = 2), each with three replicates, with standard deviation. *a* *ct* and *cox5* transcripts, encoding actin and cytochrome c oxidase subunit V, were used as internal references to normalize RNA in each reaction. SD, standard deviation. Values in parentheses were considered too variable to be used in the analysis as the standard deviation was greater than the mean. See Table 1 for the gene ID

Almost all 27 Asp-4 genes analyzed by qRT-PCR that showed increased expression during growth on sclerotial material also showed increased expression during growth on PDA. The exceptions were 1,4-ß-D-glucan cellobiohydrolase A, where no expression was detected, and pectin lyase F where expression declined over time during growth on both PDA and the sclerotial material (Tables 1 and 2). Expression of these genes tended to peak at 36 h during growth on PDA while expression of all genes was greatest at 48 h during growth on sclerotial material. The only genes that showed increased expression during growth on PDA that did not have peak levels of expression at 36 h were the 30 kDa heat shock protein, ABC multidrug transporter, fungal specific transcription factor domain protein, and transcription factor TFIID complex 145 kDA subunit genes.

### Differential protein production by *Aspergillus aculeatus* Asp-4 during growth on sclerotia of *S. sclerotiorum* and on PDA

**Fig. 1:**
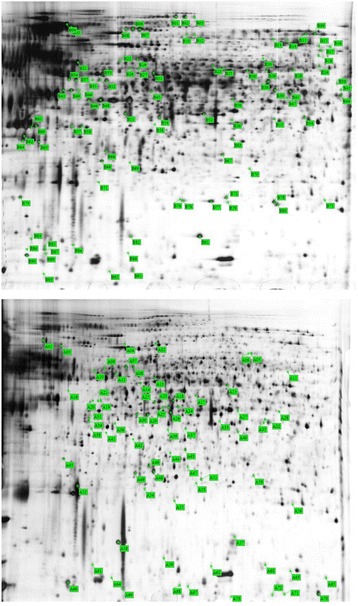
2-D gel images of intracellular protein from *Aspergillus aculeatus* Asp-4. First dimension was carried out by IEF at pH 4–7. Second dimension was done in 12.5% SDS-PAGE. Numbered protein spots were differentially accumulated in mycelia of Asp-4 grown on sclerotia of *Sclerotinia sclotiorum* versus PDA and were processed for MALDI-TOF/TOF-MS-MS analysis and protein identification

In a parallel analysis, a comparison of proteomic profiles of Asp-4 grown on *S. sclerotiorum* sclerotial material for 48 h with that of Asp-4 grown on the PDA control was performed. Data generated from two experiments were used. There were 116 protein spots that exhibited differential accumulation between these two treatments; 93 proteins that were up-regulated in the treatment where Asp-4 was grown on sclerotial material and 73 proteins that were down-regulated relative to growth on PDA (Fig. 1). MALDI TOF/TOF MS-MS analysis resulted in the identification of 33 proteins (Table 3). For the 26 identified up-regulated proteins, 18 were predicted to have different functions. Six of the down-regulated proteins were identified by MALDI TOF/TOF analysis (Table 4).

**Table 3:** *Aspergillus aculeatus* Asp-4 proteins up-regulated during colonization of *Sclerotinia sclerotiorum* sclerotial material identified with MALDI TOF/TOF MS

Spot number	Accession number	Protein homology	Molecular mass	pI	Coverage	Score	Fold change
B04	GAQ44971	*Aspergillus niger* vacuolar transporter chaperone 4.	79.5	4.69	11	313	3.60897
B06	Q9UUZ4	*A. niger* α-galactosidase C; AltName: melibiase	79.0	4.91	13	275	5.47248
B11	KJJ35102	*Aspergillus* catalase-peroxidase	83.5	5.93	18	262	3.68319
B22	KDE76293	*Aspergillus oryzae* alpha tubulin	50.1	4.94	25	171	induced
B26	ACO35262	*Aspergillus clavatus* tubulin ß-chain	50.0	4.85	18	212	induced
B27	GAQ44092	*A. niger* ß-glucosidase 1B	54.4	5.22	30	260	induced
B29	KMY30550	*Pseudomonas putida* dihydrolipoamide dehydrogenase	54.9	6.00	13	127	3.47691
B30	DLDH2_PSEPU	*Pseudomonas putida* dihydrolipoamide dehydrogenase	50.0	5.93	12	135	4.9281
B32	WP047290153, KIR22521	*Pseudomonas fluorescens* ATP synthase subunit beta	49.4	4.92	37	203	induced
B33	WP047290153, KIR22521	*P. fluorescens* ATP synthase subunit beta	49.4	4.92	37	230	15.6543
B48	XP_750867.2	*Aspergillus kawachii* class V chitinase C	43.2	4.94	8	79	5.17274
B49	GAA88849	*A. kawachii* class V chitinase	43.2	4.94	19	62	8.23482
B50	XP_001391510	*A. niger* isovaleryl-CoA dehydrogenase 2	47.0	6.57	18	110	induced
B52	Q9SV68	*Arabidopsis thaliana* putative quinone-oxidoreductase homolog	35.8	5.66	22	155	induced
B53	XP_001817513	*A.oryzae* 2,3-dihydroxybenzoic acid decarboxylase	39.0	5.32	16	210	6.14129
B60	KEY77925, XP_753324, EAL91286, AAB07619	*Aspergillus fumigatus* aspergillopepsin F	41.4	5.05	19	172	induced
B61		*A. fumigatus* aspergillopepsin F	41.4	5.05	30	314	induced
B62		*A. fumigatus* aspergillopepsin F	41.4	5.05	25	218	6.82982
B64		*A. fumigatus* aspergillopepsin F	41.4	5.05	25	172	3.96381
B68	GAA91284	*A. kawachii* HAD superfamily hydrolase	27.1	5.00	9	139	8.0873
B70	GAA83245	*Aspergillus kawachii* transcription initiation factor subunit	26.1	5.64	17	74	induced
B72	BAL22280	*Aspergillus niger* lipase	31.0	4.89	15	227	induced
B73	WP_046722239	*Pseudomonas syringae* outer membrane porin	36.7	4.69	9	156	induced
B78	BAL22280	*Aspergillus niger* lipase	31.0	4.89	17	256	47.6228
B80	BAL22280	*Aspergillus niger* lipase	31.0	4.89	17	253	8.61288
B91	XP_755466	*A. fumigatus* V-type ATPase F subunit	13.7	5.32	51	224	induced

**Table 4:** *Aspergillus aculeatus* Asp-4 proteins down-regulated during colonization of *Sclerotinia sclerotiorum* sclerotial material identified with MALDI TOF/TOF MS

Spot number	Accession number	Protein homology	Molecular mass	pI	Coverage	Score	Fold change
A02	CBF80914.1	*Emericella nidulans* 70 kDa Heat shock protein	71.0	5.57	11	84	Completely suppressed
A14	GAQ46159	*Aspergillus niger* ATP-dependent RNA helicase	43.3	5.43	27	78	Completely suppressed
A32	GAQ43185	*A. niger* class II aldolase/ adducin domain protein	30.4	5.64	26	200	Completely suppressed
A43	XP_001268906	spermidine synthase	31.4	5.52	35	461	Completely suppressed
A45	GAA92032	*Aspergillus kawachii* NADPH-dependent FMN reductase	24.9	4.65	16	124	5.58
A49	GAQ44357	*A. niger* peptidyl-prolyl cis-trans isomerase	18.8	9.02	37	64	Completely suppressed

Twelve of these up-regulated proteins potentially played roles in degradation of sclerotial material and uptake of nutrients (Table 3). These included chitinase (spot #B48, #B49), aspartic protease (aspergillopepsin F; spot # B60, #B61, #B62, #B64), and lipase (spot # 72, #78, #80) which could target the chitin, glycoprotein, and lipid components of sclerotia, respectively. ß-glucosidase (spot #B27), α-galactosidase (spot # B06) and an outer membrane porin (spot #B73) were also up-regulated. β-glucosidases hydrolyze cellobiose and other short cello-oligosaccharides to glucose while α-galactosidase hydrolyzes the terminal alpha-galactosyl moieties from glycolipids and glycoproteins. Porins play important roles in nutrient uptake and osmoregulation [52]. Notably, accumulation of the proteins ß-glucosidase, the aspartic protease Aspergillopepsin F (spot #B60, #B61), lipase (spot #B72), and the outer membrane porin (spot #B73) by Asp-4 was unique to growth on sclerotial material; being completely absent during growth on PDA.

A second major function identified in up-regulated proteins was energy metabolism (Table 3). Two up-regulated ATP synthase subunits (spot #B32, #B33), one ATPase subunit (spot #B91), and two dihydrolipoamide dehydrogenases (spot #B29, #B30) were identified. Accumulation of one ATP synthase (spot #B32) by Asp-4 was unique to growth on sclerotia. Accumulation of the ATP’ase (spot #B91) was also unique to growth on sclerotia.

A third major group of up-regulated proteins contained predicted functions that could play roles in managing environmental stress (Table 3). These up-regulated proteins were predicted to be a catalase/peroxidase (spot #B11), quinone-oxidoreductase (spot #B52), dihydrobenzoic acid decarboxylase (spot #B53), and a HAD superfamily hydrolase (spot #B68). Accumulation of the catalase-oxidoreductase was unique to growth by Asp-4 on sclerotial material. Catalase/peroxidases are bifunctional haem *b*-containing peroxidases with strong catalase activity and considerable peroxidase activity [53].

Six proteins were identified that accumulated in greater quantity in hyphae of Asp-4 grown on PDA than hyphae of Asp-4 grown on sclerotial material (Table 4). Notable protein spots were a potential HSP70 Super Family heat shock protein (spot #A02), a potential ATP-dependent RNA helicase (spot # A14), and a potential spermidine synthase (spot #A43). All were not detected in hyphae of Asp-4 grown on sclerotial material (1,000,000-fold less accumulation than on PDA). HSP70 proteins are stress response proteins expressed in the presence of biotic and abiotic stress [54–56].

### Validation of proteome results using qRT-PCR

We analyzed via qRT-PCR 13 differentially expressed proteins (9 up-regulated proteins and 4 down-regulated proteins) to validate our quantitative data from the proteomic analysis (Table 5). Two of 4 proteins predicted to be down-regulated by proteomic analysis were up-regulated in the qRT-PCR analysis. The discrepancies were with the potential RNA helicase (spot #A14) and the potential NADPH-dependent FMN reductase (spot #A45). Eight of 9 proteins predicted to be upregulated by proteomic analysis were up-regulated in the qRT-PCR analysis. The discrepancy was with the potential catalase-peroxidase (spot #B11). In all, qRT-PCR analysis was in agreement with the proteomic analysis with regard to up- or down-regulation 77% of the time. Protein accumulation has been shown to vary from gene expression in certain instances [57, 58].

**Table 5:** RT-PCR verification of up- and down-regulation of proteins

Spot	Putative function	Relative expression	
		Sclerotia	PDA
A14	Translation initiation factor 4F	3.18 ± 0.10	1.00 ± 0.01
A43	Spermidine synthase	0.38 ± 0.04	1.00 ± 0.10
A45	NADPH-dependent FMN reductase	8.98 ± 0.08	1.00 ± 0.03
A49	Peptidyl-prolyl cis-trans isomerase	0.46 ± 0.05	1.00 ± 0.05
B04	Vacuolar transporter chaperone	11.26 ± 0.04	1.00 ± 0.36
B06	α-Galactosidase	8.17 ± 0.01	1.00 ± 0.15
B11	Catalase-peroxidase	0.13 ± 0.8	1.00 ± 0.07
B22	Alpha tubulin	1.46 ± 0.06	1.00 ± 0.07
B26	Tubulin beta chain	40.70 ± 0.09	1.00 ± 1.26
B27	ß-Glucosidase 1B	120.50 ± 0.06	1.00 ± 1.65
B52	Quinone oxidoreductase	2.61 ± 0.06	1.00 ± 0.22
B53	Dihydrobenzoic acid decarboxylase	12.82 ± 0.13	1.00 ± 0.33
B68	HAD superfamily hydrolase	20.00 ± 0.06	1.00 ± 1.26

Gene expression levels were calculated from the threshold cycle according to the 2^-ΔΔCT^ method with samples from *Aspergillus aculeatus* Asp-4 grown on autoclaved, ground sclerotia of *S. sclerotiorum* and PDA. Values are the mean of two experiments (*n* = 2) each with three replicates per treatment with standard deviation. *a* *ct* and *cox5* transcripts, encoding actin and cytochrome c oxidase subunit V, were used as internal references to normalize RNA in each reaction

## Discussion

This is the first study to our knowledge where transcriptomic, qRT-PCR, and proteomic approaches were used to study the mycoparasitic interaction between an *Aspergillus* sp. and sclerotia of *S. sclerotiorum*. As with other mycoparasites of *S. sclerotiorum*, *Aspergillus aculeatus* Asp-4 expressed a diverse collection of genes, or up-regulated enzymes, potentially functioning in the cleavage of the complex set of linkages within and between polysaccharide, glycoprotein, and lipid compounds comprising the sclerotium [24, 28]. It is readily apparent from molecular studies of these disparate mycoparasites that cooperative interaction between an assortment of enzymatic capabilities is needed to degrade the physical barrier posed by the interwoven mesh of polymers comprising sclerotia, allowing penetration by the mycoparasite and conversion of sclerotial components to nutrients. *A. aculeatus* Asp-4 up-regulated expression of genes for, or produced, chitinases, aspartic proteases (aspergillopepsin F), mannan endo-1,4-β-mannosidase A, α-galactosidase, and lipases. Up-regulation of genes for, or production of these enzymes, has been detected in other mycoparasitic interactions [24, 44, 59]. Detection of genes or enzymes functioning in the degradation of sclerotial ß-1,3 or ß-1,6 glucan was notably absent from these analyses with Asp-4. However, culture filtrates from *A. aculeatus* Asp-4 growing on sclerotial material of *S. sclerotiorum* were shown to contain laminarinase activity in a prior study [30]. Laminarin is a linear β-1,3 glucan containing β-1,6 linkages. Expression of enzymes by Asp-4 capable of functioning in the degradation of melanin was also not detected during colonization of sclerotia.

It is interesting that Asp-4 up-regulated expression of genes for rhamnogalacturonate lyase A, endo-α-1,4 polygalactosaminidase, and endo-arabinase during colonization despite having no known substrate for these enzymes described within sclerotial material. Likewise, expression of genes for endoglucanases, 1,4-ß-D-glucan cellobiohydrolases, and a ß glucosidase was detected during growth of Asp-4 on sclerotia. Expression of these β-1,4-glucanases allows growth on β-1,4-glucan polymers but β-1,4 -glucan polymers such as cellulose have not been found in cell walls of sclerotia. This was also the case for *C. minitans* during mycoparasitism of sclerotia of *S. sclerotiorum* [24]. ESTs with putative gene annotations of endo-1,4-ß-xylanases and rhamnosidase A were detected despite xylan and rhamnose polymers having never been detected in sclerotia. Additionally, one or more β-1,4 glucanase, cellobiohydrolase, and β-glucosidase genes were expressed by *C. minitans* during growth on sclerotia of *S. sclerotiorum* [24]. It is possible that these carbohydrate depolymerases act as functional homologues to enzymes capable of degrading sclerotial compounds, that the appropriate corresponding polymers are present in sclerotia but have yet to be identified, or that they have the potential to function during saprophytic growth on plant residues and are co-regulated with enzymes that specifically target sclerotial components. Plant cell walls consist of polymers of rhamnose, galacturonic acid, arabinose, and xylose moities [60]. It should be noted that the cellophane used to cover the sclerotial material in petri dishes in our experiments reported here contained cellulose acetate suggesting that expression of ß-1,4-glucanases may have been due, at least in part, to cellulosic compounds in the cellophane.

Another similarity with other mycoparasites of sclerotia of *S. sclerotiorum* [24, 61] was the co-regulation or co-production of Asp-4 genes or proteins potentially functioning in adapting to environmental stress conditions with the production of these sclerotia-degrading enzymes. Genes for the heat shock proteins trehalose synthase and HSP30 were up-regulated with Asp-4 during the first 48 h of colonization of sclerotial material. Trehalose functions in thermal and oxidative stress protection [62, 63]. The 30 kDa heat shock protein HSP30 (AFU6G06470) plays a role in transcription regulation under heat shock conditions and higher expression of Hsp30 was observed both under heat stress and in response to pH stress [64]. A protease inhibitor gene was also up-regulated. Synthesis of protease inhibitors by the mycoparasite *Aspergillus niger* was postulated to be an adaptive stress response [65]. Additionally, Asp-4 up-regulated genes for, or produced, oxylate decarboxylase, quinone-oxidoreductase, dihydrobenzoic acid decarboxylase, and a HAD superfamily hydrolase; these enzymes potentially functioning in detoxification of compounds in the sclerotial environment. Disruption of an oxalate decarboxylase gene in *C. minitans* reduced the ability of this mycoparasite to infect *S. sclerotiorum* [61]. Oxalic acid is toxic to most organisms and degradation of oxalic acid by oxylate decarboxylase was thought to eliminate the toxic effect of this chemical and raise the pH making for a more beneficial environment for mycoparasitism [61, 66]. It has been postulated that reactive oxygen scavenging enzymes play key roles in oxidative and environmental stress responses in the mycoparasite *C. minitans* [24]. Several oxidoreductases, including quinone-oxidoreductase, were induced under osmotic shock of *T. harzianum*. They may counteract oxidative stress, provide NADPH for detoxification, or be involved in metabolism of oxidized molecules during metabolism [67]. Dihydrobenzoic acid has antifungal activity [68] and dihydrobenzoic acid decarboxylase may detoxify this compound. The HAD superfamily hydrolase has several functions including detoxification [69]. It should be noted that up-regulation of these genes/proteins was in response to growth on autoclaved material and not to an active response of the sclerotium to the presence of Asp-4.

The qRT PCR time course experiments with Asp-4 grown on sclerotial material and on PDA showed similarities in gene expression on these substrates (Tables 1 and 2). Almost all Asp-4 genes that showed increased expression during growth on sclerotial material also showed increased expression on PDA. The fact that both substrates contained complex mixtures of polymers including polysaccharides and proteins possibly explains this. However, the peak level of expression of genes during growth on sclerotial material tended to be at 48 h while the peak level of expression tended to be at 36 h on PDA. A possible explanation for this could be more rapid growth on PDA than the sclerotial material allowing the achievement of stationary phase more rapidly with concomitant earlier peak gene expression. PDA is a rich medium containing readily available nutrients such as dextrose in addition to mixtures of polymers while nutrients in the complex interwoven polymeric fabric of the sclerotial material may have been released more slowly. Genes for heat shock trehalose synthase, the 30 kDa heat shock protein, and oxylate decarboxylase were also all up-regulated, although to different extents, during growth on PDA and on sclerotial material. This suggests that a portion of the Asp-4 environmental stress response was general in nature being activated by different environments/substrates.

## Conclusions

In addition to substantiating parallels with other mycoparasitic interactions this study has provided the basis for molecular characterization of a previously uncharacterized mycoparasite-sclerotial interaction and may have identified previously uncharacterized genes and proteins functioning in mycoparasitism of sclerotia of *S. sclerotiorum*. Finally, the qPCR time course experiment comparing gene expression during growth of Asp-4 on sclerotial material with that of Asp-4 during growth on PDA indicates that care must be taken when interpreting transcriptomic or proteomic data where PDA was used as the control substrate. Growth on PDA resulted in up-regulation of many genes shown to be up-regulated during growth on sclerotia of *S. sclerotiorum*.

## Additional files

Additional file 1: Table S1. Primers for time course experiment. (DOCX 19 kb)

Additional file 2: Table S2. Primers for validation of proteomics. (DOCX 16 kb)

Additional file 3: Figure S1. Length distribution of unigenes and transcripts, and functional classification of unigenes. **A.** Length distribution of unigenes and transcripts of *Aspergillus aculeatus* Asp-4 grown on sclerotia of *Sclerotinia sclerotiorum*. **B.** Functional classification of unigenes of *Aspergillus aculeatus* Asp-4 grown on sclerotia of *Sclerotinia sclerotiorum* for biological process, cellular component, and molecular function. (PPTX 249 kb)

Additional file 4: Figure S2. KEGG classification of unigenes. KEGG classification of unigenes of *Aspergillus aculeatus* Asp-4 grown on sclerotia of *Sclerotinia sclerotiorum*. KEGG pathway grouping: A, cellular processes; B, environmental information processing; C, kinetic information processing; D, metabolism; E, organismal systems. (PPTX 369 kb)

Additional file 5: Table S3. Up-regulated genes vs CK. (XLS 738 kb)

Additional file 6: Table S4. Down-regulated gene vs CK. (XLS 592 kb)

## Abbreviations

Asp-4: *Aspergillus aculeatus* Asp-4
PDA: Potato dextrose agar
PDB: Potato dextrose broth
