# Supplementary material for: Characterization of mechanisms underlying degradation of sclerotia of Sclerotinia sclerotiorum by Aspergillus aculeatus Asp-4 using a combined qRT-PCR and proteomic approach
Source: BMC Genomics. 2017 Aug 31;18:674. doi: 10.1186/s12864-017-4016-8 (PMC5580281; doi:10.1186/s12864-017-4016-8)
Supplement: Supplementary file 1 — Primers for time course experiment. (DOCX 19 kb) [file 12864_2017_4016_MOESM1_ESM.docx]

Table S1. Primers used in qRT-PCR time course experiments.

______________________________________________________________________________

**Primer name** **Primer sequence** **Primer length**

**______________________________________________________________________________**

| comp14528_C0F | CCGGCTCATCAACTTCCATG | 20 |  |  |
| --- | --- | --- | --- | --- |
| comp14528_C0R | AGGAGGACGAGGAGGAGATT | 20 |  |  |
| comp15130_c0F | ACGGCTGAGAATTGTCCTCA | 20 |  |  |
| comp15130_C0r | AGCCCGGATTCAACTGTACA | 20 |  |  |
| comp15147_C0F | TTCATCCTCTTCGTCGGCTT | 20 |  |  |
| comp15147_C0R | AACACCTCAAGACGCTCAGT | 20 |  |  |
| comp15729_C0F | CCATTTTGCCTCACTTCGCT | 20 |  |  |
| comp15729_C0R | CATCCTCCTTAAAACGCGGG | 20 |  |  |
| comp16275_C7F | CGAGGGAGCGATATGTGGAT | 20 |  |  |
| comp16275_C7R | CCTGTTCCGCTATGTTCACG | 20 |  |  |
| comp7059_C0F | GCATTACTTTGGGGCTAGTCG | 21 |  |  |
| comp7059_C0R | CGAGTGCGTGGGTTTTCTTT | 20 |  |  |
| comp13560_C0F | CCGTTGTCCACTATCCGAGA | 20 |  |  |
| comp13560_C0R | GTCTAAGAGAGCGAGGGTCC | 20 |  |  |
| comp17224_C0F | TGTTCTTGTGGTTCTTGCCG | 20 |  |  |
| comp17224_C0R | GCGAGTACCACAACTTCGAC | 20 |  |  |
| comp15840_C0F | ACTCGTAGCCAAGATCCTCG | 20 |  |  |
| comp15840_C0R | GTCTCCGCATCATGACGAAC | 20 |  |  |
| comp10309_C0F | ACATCCAGAGCACAGAACGA | 20 |  |  |
| comp10309_C0R | TTGAAGAGCTGATGGAGGGG | 20 |  |  |
| comp10369_C0F | CGCGAAGGTGTAGTAGTCCT | 20 |  |  |
| comp10369_C0R | ACAAGTTCTCCCTCATCGGC | 20 |  |  |
| comp15820_C0F | CCACTTCTCTCCGTCGATCA | 20 |  |  |
| comp15820_C0R | GGTGACGACATTCTTCCACG | 20 |  |  |
| comp15955_C0F | GCAAACCACCGGAAATGACT | 20 |  |  |
| comp15955_C0R | CCTGCTTGGTTCTTGAGACG | 20 |  |  |
| comp19032_C0F | AGATCTTCGACAAAACGCCG | 20 |  |  |
| comp19032_C0R | GCGGGAGTCTTGTTGATACG | 20 |  |  |
| comp6428_C0F | CTAACGGCTTCATCTCCCCT | 20 |  |  |
| comp6428_C0R | GTAGCTGATGACGGGACCAT | 20 |  |  |
| comp7543_C0F | GACTTCTACACCAACACCGC | 20 |  |  |
| comp7543_C0R | CAGTTGTACAGGACCGAGGT | 20 |  |  |
| comp16974_C0F | ACACATCGAGCTCTACCAGG | 20 |  |  |
| comp16974_C0R | CGAGCCATGCGGATTGTAAT | 20 |  |  |
| comp16806_C0F | GCGTCTCCAACCTTTGTGTT | 20 |  |  |
| comp16806_C0R | TGTTCTGTGGCGAGTCTGAT | 20 |  |  |
| comp22183_C0F | TCCACCACCCACTGTACTTC | 20 |  |  |
| comp22183_C0R | GAGGGAGGAATGTACACGGT | 20 |  |  |
| comp10792_C0F | AGGAAAGCTTCGTCTGGTCA | 20 |  |  |
| comp10792_C0R | TACCACTTGATCCCAGGCTC | 20 |  |  |
| comp15535_C0F | AGGAAAGCTTCGTCTGGTCA | 20 |  |  |
| comp15535_C0R | TACCACTTGATCCCAGGCTC | 20 |  |  |
| comp16375_c2-F | CGATTCTATCCAAGCGCGAG | 20 |  |  |
| comp16375_c2-R | TTCACCCGTTTCCCCTTACA | 20 |  |  |
| comp19016_c0-F | CATTCTGGTGATTGAGCCCG | 20 |  |  |
| comp19016_c0-R | GTTGGTGCATTCGAGGTAGG | 20 |  |  |
| comp17477_c0-F | GGTACCCTGACTGAGATCCG | 20 |  |  |
| comp17477_c0-R | GTGTCGCCAAACAAGGTCTT | 20 |  |  |
| comp10145_c0-F | GGAGAAGAGACCTACGACGG | 20 |  |  |
| comp10145_c0-R | CCTTCAGGCTCCCTCGTTAA | 20 |  |  |
| comp12975_C0F | CTCGACCATTTCCTCCACCT | 20 |  |  |
| comp12975_C0R | TCATCCTCGACCTCAACCTC | 20 |  |  |
| comp13421_c0-F | CAGTTCATCAAGGGCAGCTC | 20 |  |  |
| comp13421_c0-R | CTTTTGAGCCGTGCAGAAGT | 20 |  |  |

_________________________________________________________________________________________
