# Supplementary material for: Characterization of mechanisms underlying degradation of sclerotia of Sclerotinia sclerotiorum by Aspergillus aculeatus Asp-4 using a combined qRT-PCR and proteomic approach
Source: BMC Genomics. 2017 Aug 31;18:674. doi: 10.1186/s12864-017-4016-8 (PMC5580281; doi:10.1186/s12864-017-4016-8)
Supplement: Supplementary file 2 — Primers for validation of proteomics. (DOCX 16 kb) [file 12864_2017_4016_MOESM2_ESM.docx]

Table S2. Primers used for qRT-PCR verfication of proteomics.

_____________________________________________________________________

**Spot** **Primer sequence** **Primer length**

**____________________________________________________________________**

| A14 F1 | GGTTTTCCCTGCCCCATTTT | 20 |  |  |
| --- | --- | --- | --- | --- |
| A14 R1 | TAGAGTCGTAGGGGTTGGGA | 20 |  |  |
| A43 F2 | CTTCGAGTTCCTCAAGCAGC | 20 |  |  |
| A43 R2 | GAGGAGCTCGAAGTAAGGCT | 20 |  |  |
| A45 F1 | CGATTTTGGGTCAGTAGGCG | 20 |  |  |
| A45 R1 | ATGGAAGCGCATGATCAACC | 20 |  |  |
| A49 F1 | AATGTTCTCCCGAAGCCTGA | 20 |  |  |
| A49 R1 | AAACGGGGATGGAGAAGAGG | 20 |  |  |
| B04 F2 | GCTGGCCAAATACTACACCG | 20 |  |  |
| B04 R2 | TGGATTCGTTTGCCTTTGGG | 20 |  |  |
| B06 F2 | GCAGAGACTTGGAATCGCTG | 21 |  |  |
| B06 R2 | GTTCTCGATGTCCTGGGTCA | 20 |  |  |
| B11 F1 | TGGCCCATCAAGCAGAAGTA | 20 |  |  |
| B11 R1 | GTTGTCTCCCCACCCCAATA | 20 |  |  |
| B22 F1 | ACATCACCACGGTAGAGCAA | 20 |  |  |
| B22 R1 | TCATCTCCGTTGACAAGGCT | 20 |  |  |
| B26 F2 | CGAGGCTCTGTACGACATCT | 20 |  |  |
| B26 R2 | AAGTGGAGACGAGGGAAAGG | 20 |  |  |
| B27 F2 | AAATACGCCCCATGAACTGC | 20 |  |  |
| B27 R2 | AATCTTTGGGGAGGGTGGAG | 20 |  |  |
| B52 F2 | CGCGTAGATTTCGTCACCTG | 20 |  |  |
| B52 R2 | CTTTGGCCCAAGAACTTCCC | 20 |  |  |
| B53 F2 | CATGCATGACCCCGAAGAAG | 20 |  |  |
| B53 R2 | TCTGCCAGAAGATGTCCCAG | 20 |  |  |
| B68 F1 | GTGATTCGAAGCAGCAGGAG | 20 |  |  |
| B68 R1 | ATCATGTCAAGGCCCAGGAA | 20 |  |  |

_______________________________________________________________________________
