# Supplementary figures and images for: Characterization of mechanisms underlying degradation of sclerotia of Sclerotinia sclerotiorum by Aspergillus aculeatus Asp-4 using a combined qRT-PCR and proteomic approach
Source: BMC Genomics. 2017 Aug 31;18:674. doi: 10.1186/s12864-017-4016-8 (PMC5580281; doi:10.1186/s12864-017-4016-8)

## Slide 1
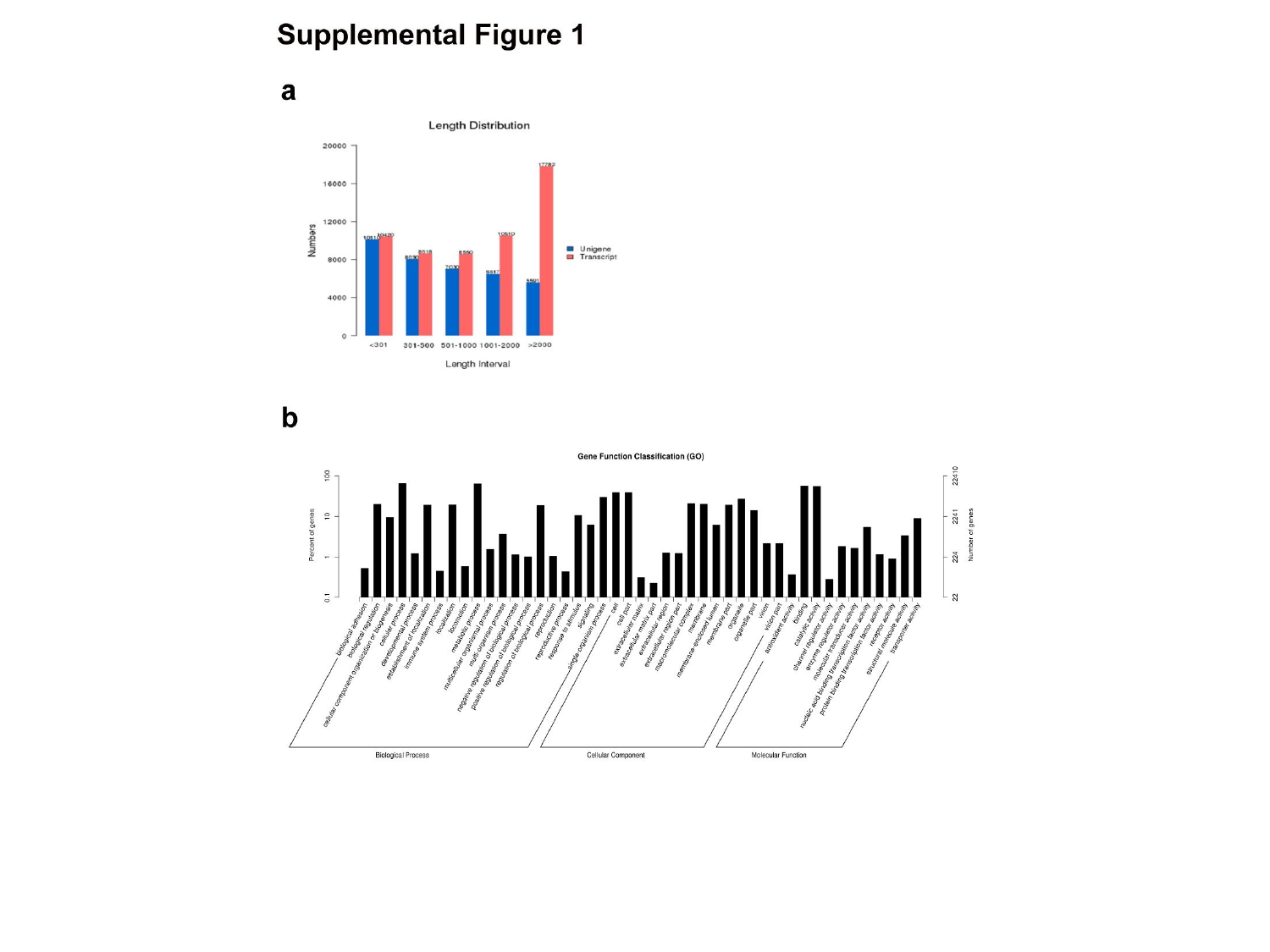

Supplement: Supplementary file 3 — Length distribution of unigenes and transcripts, and functional classification of unigenes. A. Length distribution of unigenes and transcripts of Aspergillus aculeatus Asp-4 grown on sclerotia of Sclerotinia sclerotiorum. B. Functional classification of unigenes of Aspergillus aculeatus Asp-4 grown on sclerotia of Sclerotinia sclerotiorum for biological process, cellular component, and molecular function. (PPTX 249 kb) [file 12864_2017_4016_MOESM3_ESM.pptx]

## Slide 1
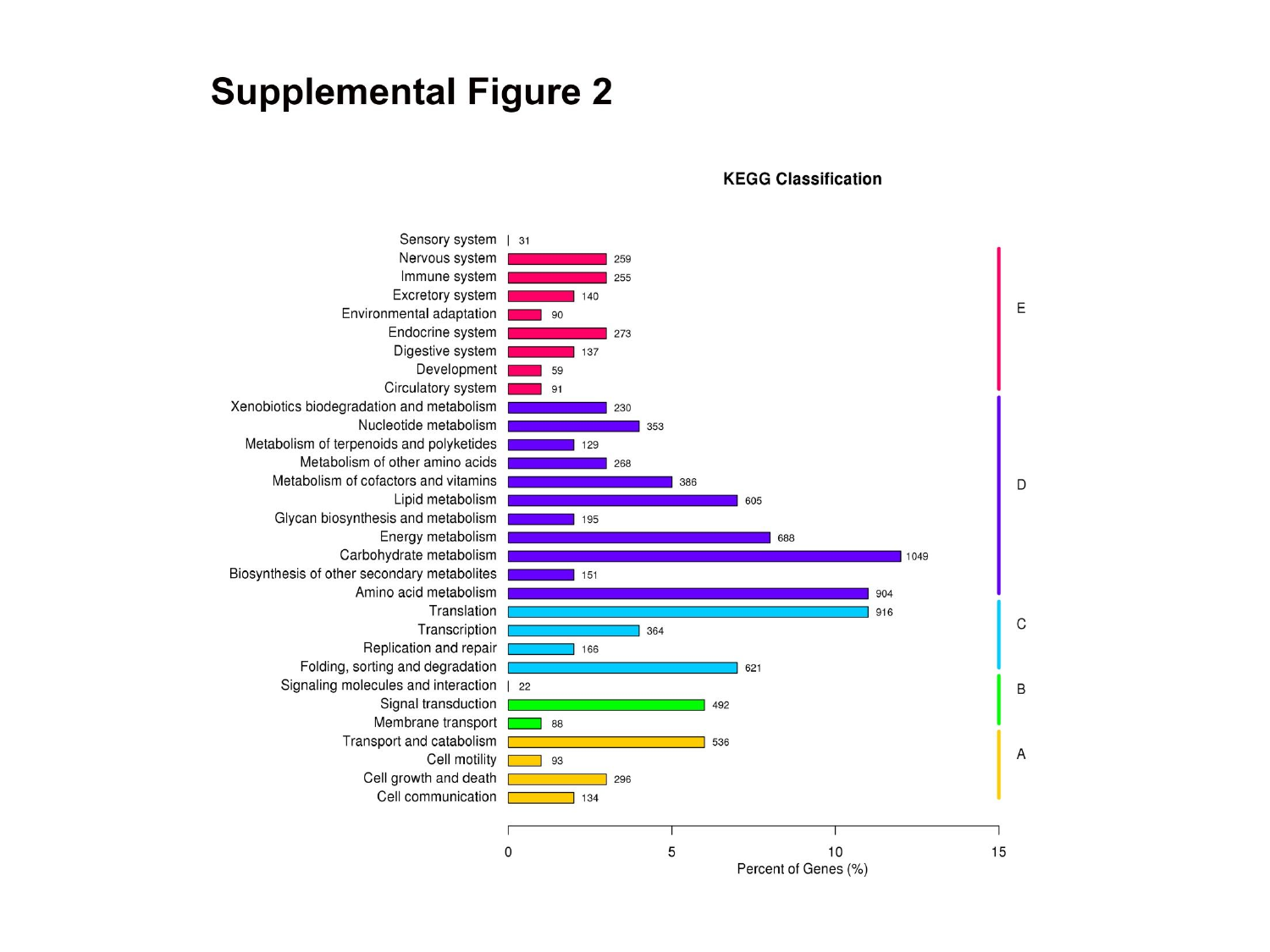

Supplement: Supplementary file 4 — KEGG classification of unigenes. KEGG classification of unigenes of Aspergillus aculeatus Asp-4 grown on sclerotia of Sclerotinia sclerotiorum. KEGG pathway grouping: A, cellular processes; B, environmental information processing; C, kinetic information processing; D, metabolism; E, organismal systems. (PPTX 369 kb) [file 12864_2017_4016_MOESM4_ESM.pptx]
